# Psychosocial health risk factors and resources of medical students and physicians: a cross-sectional study

**DOI:** 10.1186/1472-6920-8-46

**Published:** 2008-10-02

**Authors:** Edgar Voltmer, Ulf Kieschke, David LB Schwappach, Michael Wirsching, Claudia Spahn

**Affiliations:** 1 Department of Health and Behavioural Sciences, Friedensau Adventist University, An der Ihle 19, 39291 Friedensau, Germany; 2 Institute of Psychology, Psychological Diagnostics, Campus Golm, University of Potsdam, Karl-Liebknecht Str. 24-25, Potsdam, Germany; 3 Swiss Patient Safety Foundation, Asylstr. 41, Zürich, Switzerland; 4 Department of Psychosomatic Medicine and Psychotherapy, University of Freiburg, Hauptstr. 8, Freiburg, Germany; 5 Institute for Musicians' Medicine, Medical School of the University of Freiburg, Breisacher Str. 60, Freiburg, Germany

## Abstract

**Background:**

Epidemiological data indicate elevated psychosocial health risks for physicians, e. g., burnout, depression, marital disturbances, alcohol and substance abuse, and suicide. The purpose of this study was to identify psychosocial health resources and risk factors in profession-related behaviour and experience patterns of medical students and physicians that may serve as a basis for appropriate health promoting interventions.

**Methods:**

The questionnaire -Related Behaviour and Experience "Work administered in cross-sectional surveys to students in the first (n = 475) and in the fifth year of studies (n = 355) in required courses at three German universities and to physicians in early professional life in the vicinity of these universities (n = 381).

**Results:**

Scores reflecting a healthy behaviour pattern were less likely in physicians (16.7%) compared to 5^th ^year (26.0%) and 1^st ^year students (35.1%) while scores representing unambitious and resigned patterns were more common among physicians (43.4% vs. 24.4% vs. 41.0% and 27.3% vs. 17.2% vs. 23.3 respectively). Female and male responders differed in the domains professional commitment, resistance to stress and emotional well-being. Female physicians on average scored higher in the dimensions resignation tendencies, satisfaction with life and experience of social support, and lower in career ambition.

**Conclusion:**

The results show distinct psychosocial stress patterns among medical students and physicians. Health promotion and prevention of psychosocial symptoms and impairments should be integrated as a required part of the medical curriculum and be considered an important issue during the further training of physicians.

## Background

Health, stress and impairment of physicians have attracted increased attention in health care systems during the past years. Burnout rates in physicians in European and Anglo-American countries are estimated to be between 20 and 45% [1,2]. In a survey among U.S. internal medicine residents, the prevalence was as high as 76% [3]. Physicians as compared to the general population or other academic professions are also at elevated risk for other psychosocial health problems such as alcohol and substance abuse, marital disturbances or suicide [4-6]. There is increasing evidence that the development of psychosocial symptoms and impairment result from a complex interplay of several factors. Early longitudinal studies showed that personality traits in medical students evaluated before starting their course of study were important predictors for later impairment and burnout [7-9]. A highly demanding educational process and professional life frequently fostered by a competitive environment and a hierarchic culture of medicine are regarded additional relevant factors [4,10,11]. However, people respond differently to such challenges and strains. In a recent longitudinal study, Buddeberg-Fischer et al. [12] reported that while almost three quarters of evaluated Swiss physicians during residency showed no or decreasing stress levels, about 25% reported increasing or persistently high levels of stress as the extrinsic and 17% overcommitment as the intrinsic part of the effort-reward imbalance model. Individual perceptions of strain and coping styles are therefore important parameters for predicting whether health can be maintained or impairment will occur [13-16]. Research in health psychology revealed various attitudes, traits and behaviour patterns which either aggravate stress or have protecting effects as coping resources, e. g., perfectionism, compulsiveness, social support, optimism, hardiness, self-efficacy, and sense of coherence [17-19].

Tyssen et al. point out, that *types *of personality are more reliable in identifying those at risk than are *dimensions *of personality alone [20]. It has also been reported that male and female students and physicians differ in their experience and response to stress and psychosocial symptoms [21-23].

Germany has no longstanding tradition in addressing physicians' health and impairments. Resch and Hagge [24], two German psychologists, note that care for physicians' health in Germany is still at the level of the USA in the '60's. The aim of this study was therefore to investigate work-related experiences and behaviour patterns in medical students and physicians in Germany at different points in their medical education and professional life. Based on previous work by Schaarschmidt & Fischer, we aimed to identify risk factors or health resources for subsequent psychosocial impairments. We used the personality typology described by these authors derived from the cluster analysis of eleven health relevant dimensions from the domains of professional ambition, resistance to stress and emotional well-being, which comprises four health relevant patterns (the healthy pattern G, the unambitious pattern S, the overcommitted risk pattern A und the resigned risk pattern B) [25-27].

Our hypotheses were:

(1) A majority in first year students presents a healthy pattern.

(2) Physicians are less likely to present healthy patterns and more likely to present risk patterns and less favourable scores in the health relevant dimensions compared to first and fifth year students.

(3) Differences between male and female students and physicians were expected, in particular in the domains of professional ambition, resistance to stress and social support.

## Methods

### Study design

We conducted three cross-sectional surveys among medical students and physicians. In order to achieve a high response rate in students of more than one university, we recruited subjects at a teaching unit in required courses at three universities in three different federal states in Germany. Students in their first and fifth year of study were presented with the study goals and a questionnaire was administered. Participation was voluntary. The students were given sufficient time for completion and the questionnaires were collected immediately afterwards. Return after completion was deemed informed consent. There was no dependency relationship between students and researchers (e. g., evaluation, supervision). Physicians' data were gathered by means of a mail-in survey in the vicinity of the three universities. Addresses were selected with the support of the local Medical Associations or the university. Selection criteria were the number of postgraduate years in professional work-life. Because the study was associated with minimal risks and complied with the data protection rules, it was approved by the ethics commission of the University of Freiburg in a minimal risk review and exempted from a full formal evaluation.

### Response rate and demographic data

The response rates of the three cross-sectional surveys were 85.6% (n = 475) among students in the first year, 76.3% (n = 355) in the fifth year, and 31.6% (n = 381) among physicians. In the sample of physicians, responders were slightly younger (34.8 SD 4.0 vs. 35.7 SD 4.3, p < .001) and more likely to be female (48.0% men, 52.0% women vs. 52.7% men, 47.3% women, p = .274) compared to the total mail-in group. To obtain a more coherent sample, data analyses were restricted to data provided by physicians in their third to eighth year of professional work (n = 344) and responses by physicians with longer or shorter times in practice were excluded. Table 1 shows the sample characteristics of the study groups. Among students, more than 60% were female. The number of male and female responders among physicians was almost equal. The majority of physicians were married while most of the students reported living "single". Approximately 80% of physicians worked at in-patient institutions (hospitals, rehabilitation clinics), the remainder in a private practice (13.4%) or other areas (6.4%).

**Table 1:** Demographic characteristics of students and physicians

		Students 1st year n = 471	Students 5th year n = 345	Physicians 3rd–8th year n = 344
Age (SD)		20.8 (2.6)	24.6 (2.5)	34.4 (3.4)
Gender	female (%)	61.7	66.7	51.2
	male (%)	38.3	33.3	48.8
Nationality	German (%)	89.9	94.9	98.8
	other (%)	10.1	5.1	1.2
Marital status	married (%)	1.5	4.1	53.8
	with partner (%)	19.6	39.1	23.0
	single (%)	78.7	56.0	19.5
	divorced/separated/widowed (%)	0.2	0.9	3.8

### Survey instruments

In addition to a set of demographic questions, the questionnaire "Work-Related Behaviour and Experience Patterns" (AVEM)[25,26,28] was administered. The instrument has been developed to gather self-reported data about personal experiences with work-related stress and typical behavioural responses to cope with stress. In detail, the questionnaire covers the following three major domains: a) professional commitment, b) resistance to stress, and c) emotional well-being (in the context of work) which are assessed with 11 separate scales (Additional file 1). Each scale consists of 6 items with response options presented as a 5-point Likert-scale ranging from "I strongly disagree" to "I strongly agree". In order to determine an individual's pattern of behavioural response to occupational stress, it is necessary to go beyond the analysis of the single scales. Information about the configuration of the characteristics has a more predictive value than distinct parameters for each dimension [20]. For example, high professional commitment in itself does not constitute a health risk. However, if a tendency to high professional commitment is coupled with reduced capacity to cope, the combination of these two traits may put the individual at a higher risk for developing health problems. In order to identify characteristic configurations of behavioural traits that help to estimate a person's health status and risk, respectively, Schaarschmidt & Fischer [25] analysed the data of the initial AVEM sample group (N = 1,598), a group comprising representatives of diverse professions. A cluster analysis revealed a four-cluster solution. The same cluster solution was replicated with sufficient concurrence in 10 random samples drawn from the entire sample of 1,598 test subjects (average κ > 0.80) [25]. Scale reliability was assessed in samples of different professions. The median Cronbach's α as a measure of internal consistency was 0.81 (minimum: 0.79, maximum: 0.86). The validity of the measure was also supported by moderate to good correlations with scales measuring related constructs (e. g., FPI, MBI). The health relevance of the patterns was supported by expected correlations with a broad set of criteria such as depression, well-being, sickness, absence, blood pressure, heart rate, type-A behaviour or burnout.

The four different types of work-related experience and behaviour patterns derived from the cluster analysis are described as follows [25-28]:

#### 1. Type G: The 'Healthy-Ambitious' Type

This pattern represents a healthy attitude towards work. The individuals are ambitious at work, but also able to maintain emotional distance from work. They score high in the dimensions that represent resistance to stress and in all dimensions related to positive emotions.

#### 2. Type S: The 'Unambitious' Type

A rather unambitious attitude towards work is characteristic for this pattern with the lowest scores in the dimensions describing commitment to work and highest scores in capacity for detachment. Nevertheless, low scores with a tendency to resignation and medium to high scores for inner balance, satisfaction with life, and the experience of social support suggest a generally positive experience with life. The challenge of this pattern is less in health than in promoting motivation.

In contrast to patterns G and S, the following two are repeatedly shown to be linked to illness. They therefore play a key role in preventing impairment and promoting health.

#### 3. Risk Type A: The 'Excessively Ambitious' Type

This pattern is characterised by excessive commitment to work and difficulties with emotional distancing from work. Limited coping abilities in stressful situations and negative emotions also characterise this exhausting pattern. Individuals with risk pattern A show many similarities to the concept of type-A behaviour described for coronary artery disease and myocardial infarction [29]. High positive correlations have been observed for risk type A and type A behaviour.

#### 4. Risk type B: The 'Resigned' Type

Individuals with this pattern show low scores for the dimensions related to professional commitment. They attain high scores for the tendency to resignation and correspondingly low scores for emotional distancing and active coping. Their emotional status is characterised by low scores for balance and mental stability, satisfaction with work, and satisfaction with life, and shows limited experience of social support. This pattern represents the core symptoms of burnout syndrome and correlates with burnout scores measured with standard instruments (MBI) [25].

### Statistical analysis

All analyses were carried out with SPSS for Windows release 10.0 (SPSS Inc., Chicago, IL, USA). Descriptive statistics are presented in terms of counts, percentages, means and standard deviations. For hypothesis (1), the relative fractions of the different behavioural patterns were analysed. For hypothesis (2) and (3), the differences between study groups in the AVEM-dimensions were analysed in a two-factorial MANCOVA using general linear model (GLM) estimations with AVEM-dimensions as dependent variables, study groups and gender as independent variables and marital status as covariate. Samples were not adjusted for age due to the high natural correlation between age and study groups. Differences in the categorical behaviour and experience patterns were tested with χ^2 ^test. The Bonferroni method was used to adjust for multiple tests.

## Results

### Behaviour patterns

**Figure 1:**
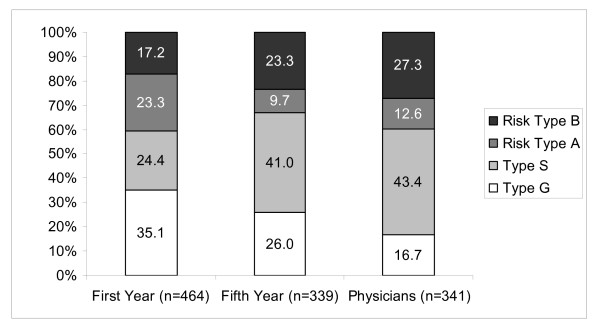
**Behaviour and experience patterns of medical students and physicians**. Type G: healthy pattern, Type S: unambitious pattern, Risk type A: pattern of overexertion, Risk type B: resignation pattern.

The distribution of risk patterns in the three samples is presented in figure 1. The highest frequency of resigned and burnout-related risk pattern B was observed in the physician sample (27%). The most common pattern in physicians and fifth year students was the unambitious type S (43% vs. 41%). In the sample of first-year students, 35% presented the healthy and ambitious pattern G, but nearly a quarter of responders showed the unambitious pattern S and the excessively ambitious risk pattern A (Figure 1). The differences between the distribution of patterns in the study groups were highly significant (χ^2 ^= 85.65; df = 6; p < .001; φ = .27). Only the fifth-year students showed differences between male and female students in the distribution of patterns χ^2 ^= 9.32; df = 3; p = .025; φ = .17), mainly due to the lower prevalence of pattern G and higher prevalence of the unambitious pattern S among female physicians.

### Health-relevant dimensions

**Figure 2:**
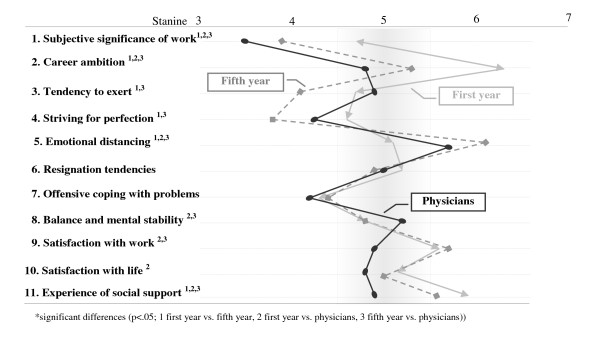
**Health-relevant dimensions of medical students and physicians**. The eleven dimensions can be divided into three domains: professional commitment (1–5), coping capacity (6–8) and emotional well-being (9–11). Results are presented as stanine scores. ("Standard nine" (Stanine) is a method of scaling test scores on a nine-point standard scale with a mean of five and a standard deviation of two and was used to compare scores of the study groups in one digit number.)

Results of the two-factorial analysis of variance show a significant difference between the study groups (Wilks λ = .76, F(22, 2246) = 15.37, p < .001, total effect size η^2 ^= .13) at the level of the dimensions controlling for gender and marital status. The first-year students differed significantly from the fifth-year students in six dimensions mainly relating to the domains professional commitment and resistance to stress. In the dimensions significance of work, career ambition, tendency to exert, and striving for perfection, the fifth-year students scored much lower, while scoring higher on emotional distancing. In the dimensions of emotional well-being, first year students presented slightly higher scores of social support than fifth year students. Physicians scored significantly lower on subjective significance of work and career ambition, and higher on emotional distancing than both student groups. The dimensions of emotional well-being (satisfaction with work and life and experience of social support) were also lower compared to students. The physicians only scored higher than the students with respect to balance and mental stability (Figure 2).

Table 2 shows the total raw scores of the study groups for the AVEM dimensions, differences between male and female subjects and main effects and interactions of independent variables and covariates. In all three samples, females scored lower on career ambition and higher on resignation tendencies. Scores for balance and mental stability in the student groups were higher in males compared to females. Among fifth year students and physicians, females reported more social support than males. The female physicians scored higher on the dimension satisfaction with life. The main effects of sample membership, gender and marital status were significant whereas the interaction between gender and study groups was barely significant (p = .049) and showed differences in only two dimensions with a weak effect size.

**Table 2:** Health-relevant dimensions in men and women

	First Year							Fifth Year							Physicians														
	Total (n = 464)		Male (n = 176)		Female (n = 288)			Total (n = 339)		Male (n = 113)		Female (n = 226)			Total (n = 341)		Male (n = 165)		Female (n = 176)			cohorts		gender		marital status		cohorts* gender	

	M	SD	M	SD	M	SD	p	M	SD	M	SD	M	SD	p	M	SD	M	SD	M	SD	p	p	eta^2^	p	eta^2^	p	eta^2^	p	eta^2^

subjective significance of work	16.29	4.76	15.78	5.21	16.60	4.45	0.053	14.33	4.40	14.37	4.88	14.31	4.15	0.808	13.19	4.59	13.17	4.68	13.20	4.52	0.856	0.000	0.045	0.325	0.001	0.071	0.003	0.350	0.002
career ambition	21.64	4.15	22.28	4.10	21.25	4.14	0.008	19.43	4.84	20.90	5.49	18.69	4.30	0.000	18.01	4.85	19.35	4.85	16.76	4.52	0.000	0.000	0.090	0.000	0.042	0.369	0.001	0.041	0.006
tendency to exert	18.28	3.95	18.35	4.04	18.23	3.90	0.675	17.00	4.49	17.84	4.77	16.58	4.30	0.012	18.84	5.19	19.20	4.96	18.51	5.39	0.191	0.000	0.018	0.012	0.006	0.777	0.000	0.228	0.003
striving for perfection	21.23	4.30	21.52	4.51	21.06	4.17	0.250	19.31	4.41	19.40	4.54	19.27	4.36	0.688	20.71	4.29	20.72	4.10	20.71	4.48	0.971	0.000	0.032	0.414	0.001	0.218	0.001	0.742	0.001
emotional distancing	17.18	3.88	17.69	3.85	16.88	3.88	0.029	19.88	3.88	19.31	3.96	20.17	3.82	0.040	18.89	5.10	18.89	5.13	18.89	5.08	0.888	0.000	0.047	0.959	0.000	0.841	0.000	0.032	0.006
resignation tendencies	16.98	4.19	15.89	3.93	17.65	4.22	0.000	16.31	4.61	15.27	4.63	16.82	4.52	0.005	16.57	4.79	15.76	4.63	17.33	4.83	0.003	0.088	0.004	0.000	0.029	0.317	0.001	0.935	0.000
offensive coping with problems	20.97	3.54	21.54	3.59	20.62	3.48	0.003	21.09	3.99	21.73	4.18	20.77	3.86	0.034	20.71	3.69	21.13	3.35	20.31	3.96	0.054	0.080	0.004	0.000	0.014	0.105	0.002	0.970	0.000
balance and mental stability	19.17	4.31	20.29	4.14	18.48	4.26	0.000	19.23	4.54	20.19	4.96	18.75	4.24	0.008	19.98	4.89	20.44	5.19	19.56	4.57	0.153	0.168	0.003	0.000	0.021	0.705	0.000	0.362	0.002
satisfaction with work	24.03	3.74	23.56	3.74	24.33	3.72	0.048	24.09	3.95	23.97	4.51	24.15	3.65	0.772	22.54	4.12	22.61	4.13	22.48	4.12	0.947	0.000	0.036	0.300	0.001	0.001	0.010	0.267	0.002
satisfaction with life	22.74	3.90	22.46	4.08	22.91	3.79	0.355	22.13	4.48	22.11	4.69	22.15	4.38	0.977	21.76	4.66	21.22	4.39	22.26	4.86	0.005	0.000	0.027	0.086	0.003	0.000	0.031	0.334	0.002
experience of social support	24.78	4.29	24.48	4.19	24.97	4.35	0.235	24.00	4.35	23.11	4.57	24.44	4.18	0.011	22.74	4.73	22.21	4.66	23.23	4.76	0.011	0.000	0.047	0.001	0.010	0.000	0.014	0.419	0.002

Total raw scores of the study groups for the AVEM dimensions, differences between male and female subjects and main effects and interactions of independent variables and covariates.

## Discussion

The main purpose of our study was to identify psychosocial health risks and resources in the study- or work-related behaviour and experience in medical students and physicians. The AVEM questionnaire identifies not only important domains and dimensions of work-related individual behaviour, but also allows the identification of patterns that indicate serious health risks.

### Health-relevant behaviour and experience patterns

The majority of first-year students displayed the healthy and ambitious pattern G. However, it is important to note that different from our first hypothesis, this was not the predominant pattern: approximately one fourth of the students presented the overambitious risk pattern A. While this may have been expected taking into account the challenging education and professional life, one has to recall that this risk pattern represents a tendency to overexert and difficulties to distance oneself from work. A comparable fraction presented with the unambitious S type, and 17% of students with a resigned and burnout-related risk pattern B are determined as early as at the beginning of their studies. This finding is supported by other studies that describe a similar degree of psychological vulnerability at this early stage of study and emphasise the importance of personality traits for the development of stress and psychosocial symptoms [20,30,31].

In concordance with our second hypothesis, physicians presented lower scores of the healthy behaviour and experience pattern than both student groups, and correspondingly higher scores for the burnout-related risk pattern B. This confirms results from other researchers who describe an accumulation and worsening of effects in medical education and professional life over time [4,32,33]. In Swiss medical residents both men and women reported a significantly worse physical and psychological well-being as well as life satisfaction after their first year of residency compared to the time directly before their graduation from medical school [21]. Schaarschmidt and Fischer [25] describe a correlation between risk pattern B and standard measurements of burnout (MBI [34], BHD [35]). Other researchers therefore applied the AVEM to assess burnout in relation to the psychopathological and psychosomatic symptom load (SCL90R). Risk pattern B showed the highest scores of psychiatric symptoms (in particular depression) in the SCL90R [36]. It seems therefore appropriate to compare the scores for resigned risk pattern B of the physicians surveyed in our study with burnout rates reported elsewhere. The prevalence of risk pattern B among physicians in our study is quite similar to estimates of burnout in Germany in general [2]. Lower scores have been reported for general practitioners in Switzerland [37], higher scores in a survey among Canadian physicians [1]. The observed fraction of the healthy pattern G among physicians is among the lowest reported for other professions such as teachers, business founders or nurses [36,38].

### High distance and demotivation in fifth year students and physicians

The most impressive result of this study is certainly the high fraction of unambitious pattern S observed in fifth-year students and physicians. In contrast to the popular stereotype of physicians being highly motivated high achievers in a challenging profession, this type is characterised by low self-perceived significance of work and professional ambition and high emotional distancing from work. Taking into account the lower values for the dimensions of emotional well-being, in particular in male physicians, this seems to reflect frustration and overwork. The high fraction of resigned risk pattern B among physicians (27%) which represents the core symptoms of the burnout syndrome supports this interpretation. While the relatively low response rate in physicians may have influenced this result, comparisons between responders and mail-in group do not indicate a strong selection bias. The low response rate of physicians might itself be an additional clue of demotivation and poorer coping. Other researchers confirm increasing demotivation even in younger physicians [39,40]. The poor coping styles observed in the physician sample therefore suggest that both primary medical education as well as continuing medical training in physicians should target the emotional well-being and health concerns relating to the profession. This is emphasised by the fact that psychiatric morbidity in students and physicians is common but students and physicians are reluctant to seek help [9,41,42].

### Gender-related differences in health-related dimensions

As hypothesised, females scored lower than males on career ambition in all study groups. Especially in fifth year students and physicians this may reflect differences in career aspirations and coping with professional experiences [43,44] as well as efforts to combine family and professional life [45,46]. It is striking that women scored lower than men particularly in the domain of resistance to stress that covers the dimensions resignation tendencies, offensive coping with problems as well as balance and mental stability. This is confirmed by other researchers who report higher scores of depression and worse mental well-being in women than in men in medical student or physician samples [47,48]. Because the majority of medical students in Germany are currently female, this issue needs to be addressed by interventions that support effective coping.

Compared to both student groups, physicians presented lower scores in the dimension experience of social support. Withdrawal from social relationships in response to educational or professional stress in medical students and physicians has been reported by others [49-51]. We observed no significant gender-related differences in first-year students relating to social support but among fifth-year students and physicians, females scored significantly higher than their male counterparts. Since social support has been shown to be one of the most important supportive factors for dealing with stress and impairment in medical students and physicians [52,53] and is even more important to women [54], this issue should be addressed in medical education and postgraduate training.

This study has also a number of limitations: firstly, we surveyed three distinct populations at different stages in their career, and thus the data are not longitudinal. While there is no indication for this, differences in patterns may be explained by the differences between populations rather than an outcome of career advancement. Secondly, the response rate among physicians is comparable to the one obtained in other studies, but remains low. We therefore cannot exclude the possibility that our results are affected by response bias. However, as there were no differences in the gender distribution and only small differences in age between responding and addressed physicians, this bias – if any – should be small.

## Conclusion

In summary, our data show a consistently lower prevalence of the healthy pattern G in fifth-year students and physicians compared to first-year students, and a higher prevalence of the resigned risk pattern B. Differences between the student samples were observed in the domains of professional ambition and resistance to stress. Women show less favourable scores in the dimensions of professional ambition and resistance to stress, but score higher on social support.

A large number of studies, including the one presented here, demonstrate the potential health risks resulting from the strains and burden associated with the medical education and profession. It seems paradoxical, that the training and professional activity aimed at helping and healing others show little awareness towards potentially harmful effects, and little willingness to engage in the prevention of a declining health in candidates of the medical profession themselves. This is essential not only for the benefit of medical students and physicians, but also for the provision of high quality care to patients.

## Competing interests

The authors declare that they have no competing interests.

## Authors' contributions

EV: idea, design, organisation of studies, acquisition, analysis and interpretation of data, writing the paper. UK: analysis and interpretation of data, critical revision of the article for important intellectual contents, approval of the final version. DS: Acquisition of data, contributions to conception and design, critical revision of the article for important intellectual contents and approval of the final version. MW: substantial contributions to conception and design, critical revision of the article for important intellectual contents and approval of the final version. CS: idea, design, organisation of studies, data acquisition, critical revision of the article for important intellectual contents and approval of the final version.

## Pre-publication history

The pre-publication history for this paper can be accessed here:



## Supplementary Material

Additional file 1 Appendix Click here for file
