# Supplementary material for: Psychosocial health risk factors and resources of medical students and physicians: a cross-sectional study
Source: BMC Med Educ. 2008 Oct 2;8:46. doi: 10.1186/1472-6920-8-46 (PMC2567308; doi:10.1186/1472-6920-8-46)
Supplement: Additional file 1 — Appendix [file 1472-6920-8-46-S1.pdf]

Work-related Behaviour and Experience Pattern (AVEM)

U. Schaarschmidt & A. W. Fischer

name or code: .... sex: ....

profession: .... age: ....

In the following questionnaire you are requested to describe some of your normal behavioral traits, attitudes and habits, particularly in regard to your work. These are formulated as a list of statements.

Please indicate to what extent these statements apply to you personally:

No.	Statement	I strongly agree	I somewhat agree	I'm in the middle	I somewhat disagree	I strongly disagree
		●	◕	◑	◔	○
1.	Work is the most important element in my life.	●	◕	◑	◔	○
2.	I want to achieve more in my career than most people I know.	●	◕	◑	◔	○
3.	If necessary, I will work until I am exhausted.	●	◕	◑	◔	○
4.	My work should never contain errors or deficiencies.	●	◕	◑	◔	○
5.	After work is over I can forget about it quickly.	●	◕	◑	◔	○
6.	I quickly resign myself to lack of success.	●	◕	◑	◔	○
7.	For me, difficulties are there to overcome.	●	◕	◑	◔	○
8.	I don't get upset easily.	●	◕	◑	◔	○
9.	Until now I have been successful in my work.	●	◕	◑	◔	○
10.	So far, I have been satisfied with my life.	●	◕	◑	◔	○
11.	My partner shows understandig for my work[1]	●	◕	◑	◔	○
12.	My work is everything to me.	●	◕	◑	◔	○
13.	My career doesn't mean much to me.	●	◕	◑	◔	○
14.	I always give it all I've got	●	◕	◑	◔	○
15.	I prefer to check everything three times over rather than hand in work that contains mistakes.	●	◕	◑	◔	○
16.	I still go on thinking about work problems in my leisure time.	●	◕	◑	◔	○
17.	I find it difficult to cope with lack of success	●	◕	◑	◔	○
18.	If I don't succeed, I say to myself: „This time nothing will stop me!"	●	◕	◑	◔	○
19.	I am a restless person.	●	◕	◑	◔	○
20.	Up to this point in my career, I have experienced more success than disappointments.	●	◕	◑	◔	○
21.	By and large, I am happy and content	●	◕	◑	◔	○
22.	My family isn't very interested in my problems at work	●	◕	◑	◔	○
23.	I could be quite happy without my work.	●	◕	◑	◔	○
24.	As far as my career is concerned, I consider myself to be fairly ambitious	●	◕	◑	◔	○
25.	I work more than I really should.	●	◕	◑	◔	○

[1] or the person to whom you are closest

© U. Schaarschmidt & A. W. Fischer, University of Potsdam, Germany

I strongly agree ● / I somewhat agree ◕ / I'm in the middle ◐ / I somewhat disagree ◔ / I strongly disagree ○

26. One of my goals is not to make any mistakes at work.	●	◕	◐	◔	○
27. After work I can switch off and easily forget problems.	●	◕	◐	◔	○
28. Failure at work is very discouraging for me.	●	◕	◐	◔	○
29. Lack of success doesn't discourage me, but makes me try even harder next time.	●	◕	◐	◔	○
30. I consider myself to be rather hectic.	●	◕	◐	◔	○
31. So far, I haven't had great success at work.	●	◕	◐	◔	○
32. I have good reason to look into the future with optimism.	●	◕	◐	◔	○
33. I would like my partner[2] to have more consideration for my work and its problems.	●	◕	◐	◔	○
34. I need my work like the air I breathe.	●	◕	◐	◔	○
35. In terms of career, I am aiming to get further than most other people.	●	◕	◐	◔	○
36. I tend to overwork.	●	◕	◐	◔	○
37. Whatever I do, it must be perfect.	●	◕	◐	◔	○
38. Leisure time is leisure time – I don't lose any sleep over work.	●	◕	◐	◔	○
39. Failure at work makes me very depressed.	●	◕	◐	◔	○
40. I am sure that I'll be able to deal with all future challenges in my life.	●	◕	◐	◔	○
41. I think I am a calming influence on the people around me.	●	◕	◐	◔	○
42. So far, I have been very successful in my career.	●	◕	◐	◔	○
43. I have no reason at all to be dissatisfied with my life.	●	◕	◐	◔	○
44. I have the full support of my family.	●	◕	◐	◔	○
45. I don't know how I could live without my work.	●	◕	◐	◔	○
46. I have great plans for my future career.	●	◕	◐	◔	○
47. My daily routine is characterized by a chronic lack of time.	●	◕	◐	◔	○
48. I don't consider my work to be finished until I am completely satisfied with the result.	●	◕	◐	◔	○
49. Problems at work occupy my mind all day.	●	◕	◐	◔	○
50. Whenever I fail, I am easily discouraged, even when I have tried hard.	●	◕	◐	◔	○
51. Lack of success can challenge me to try harder.	●	◕	◐	◔	○
52. I can remain calm and collected in almost all situations.	●	◕	◐	◔	○
53. My life up till now has been characterized by success at work.	●	◕	◐	◔	○
54. I have been quite disappointed by some aspects of my life.	●	◕	◐	◔	○
55. Sometimes I wish I could receive more support from the people around me.	●	◕	◐	◔	○
56. There are things in life that are more important than work.	●	◕	◐	◔	○
57. Success at work is an important aim in my life.	●	◕	◐	◔	○
58. I put everything I've got into my work.	●	◕	◐	◔	○
59. I don't like having to finish work, which could be improved on.	●	◕	◐	◔	○
60. My thoughts are always circling round my work.	●	◕	◐	◔	○
61. If I have experienced some kind of failure, I can feel very discouraged.	●	◕	◐	◔	○
62. If I don't succeed in something, I don't give up, but try even harder.	●	◕	◐	◔	○
63. I remain calm in the midst of turmoil.	●	◕	◐	◔	○
64. I can feel proud of my achievements at work.	●	◕	◐	◔	○
65. There can't be many people who are happier than I am.	●	◕	◐	◔	○
66. When I need help and advice, there is always someone there.	●	◕	◐	◔	○

[2] or the person to whom you are closest
